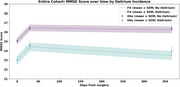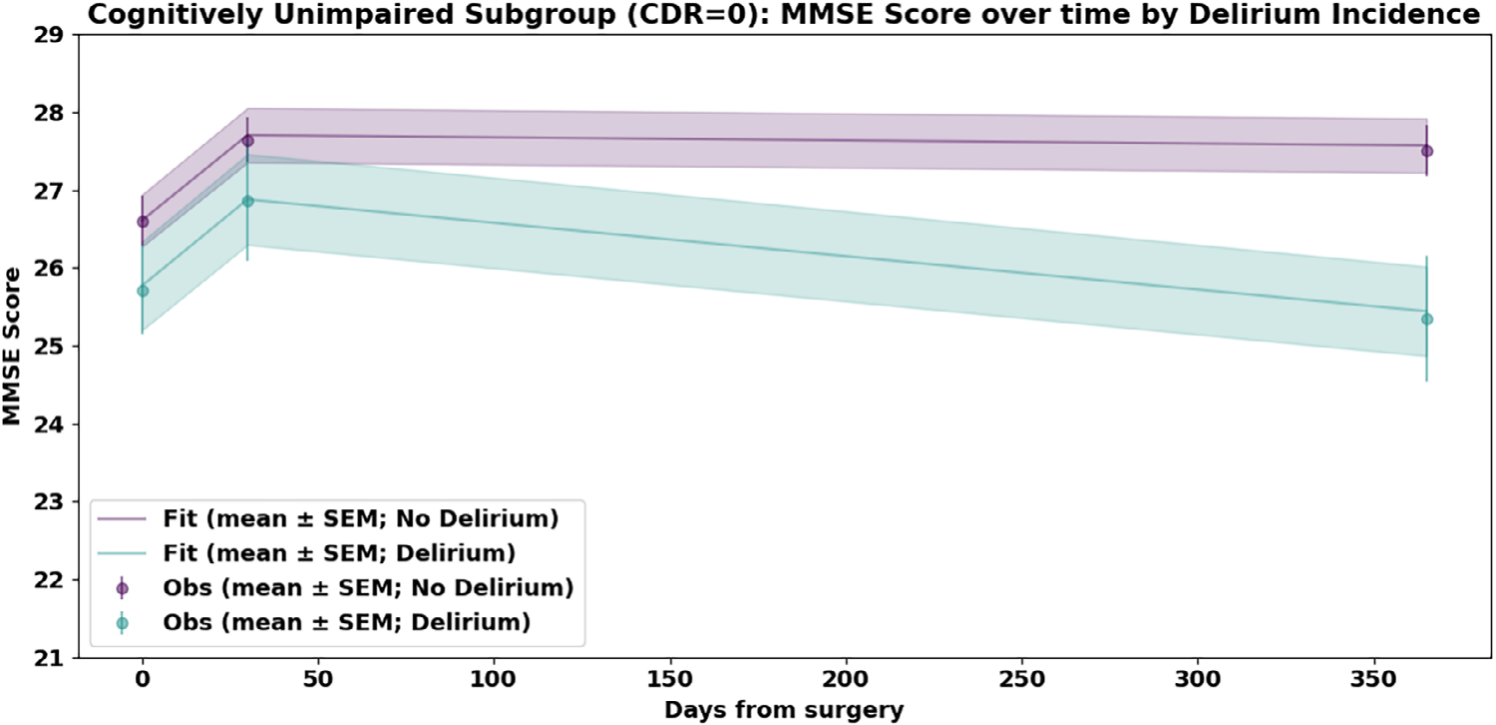# Who stands to lose the most: impact of delirium on long‐term cognitive impairment in a hip fracture repair cohort

**DOI:** 10.1002/alz70857_103842

**Published:** 2025-12-25

**Authors:** Mfon E Umoh, Anirudh Sharma, Jeannie‐Marie S Leoutsakos, Kostas G. Lyketsos, Sharon K. Inouye, Edward R Marcantonio, Paul B. Rosenberg, Karin J Neufeld, Frederick Sieber, Esther S. Oh

**Affiliations:** ^1^ Johns Hopkins University School of Medicine, Baltimore, MD, USA; ^2^ Department of Psychiatry and Behavioral Sciences, Johns Hopkins University School of Medicine, Baltimore, MD, USA; ^3^ Marcus Institute for Aging Research, Hebrew SeniorLife, Boston, MA, USA; ^4^ Beth Israel Deaconess Medical Center, Boston, MA, USA; ^5^ McMaster University, Hamilton, ON, Canada; ^6^ Johns Hopkins Medicine, Baltimore, MD, USA; ^7^ Division of Geriatric Medicine and Gerontology, Department of Medicine, Johns Hopkins University School of Medicine, Baltimore, MD, USA

## Abstract

**Background:**

Delirium, an acute disorder of attention and cognition, is a potentially preventable contributor to poor outcomes in older adults including future cognitive decline. The goal of this study was to determine the cognitive impact of postoperative delirium on patients who underwent hip fracture repair (HFR) over a 1‐year period. Our hypothesis was that the downstream impact of delirium may be greater among individuals who are cognitively unimpaired compared to those who have cognitive impairment at baseline.

**Method:**

Cognitive assessments from 200 HFR patients enrolled in the randomized clinical trial “A Strategy to Reduce the Incidence of Postoperative Delirium in Elderly Patients” (STRIDE) were examined for cognitive changes at 1‐year after surgery. Inclusion criteria were age ≥65 and Mini‐Mental State Exam (MMSE) score ≥15. Delirium status and Clinical Dementia Rating (CDR) were adjudicated by a consensus diagnostic panel. Global CDR score was used to define subgroups. Data were analyzed using a random‐intercept linear spline model, with MMSE over time as outcome of interest; MMSE scores at baseline, 1‐month, and 1‐year were used.

**Result:**

Delirium incidence in this cohort was 36.5%. Prior to surgery, 41% were cognitively unimpaired (CDR=0), while 58% had mild cognitive impairment or dementia (CDR>0), 2 cases were missing CDR. Baseline MMSE was 2.054 points lower for the group that experienced delirium compared to the no delirium group in the entire cohort (Figure 1). Delirium incidence was associated with rate of decline in MMSE scores in the entire cohort. Incident delirium was associated with more rapid rate of MMSE decline in the entire cohort (β=‐0.002, SE=0.001, *p* = 0.110) (Figure 1) and in the subgroup without cognitive impairment (CDR=0) (β=‐0.004, SE=0.002, *p* = 0.046) (Figure 2); but not in participants with baseline cognitive impairment (CDR>0). Greater delirium severity, measured by Delirium Rating Scale‐Revised‐98 (DRS‐R‐98), was associated with more rapid decline in MMSE only for the subgroup without cognitive impairment (β = ‐0.0004, SE=0.0001, *p* = 0.004).

**Conclusion:**

Delirium incidence and severity impact cognitive changes in older adults undergoing HFR, most significantly in those who are cognitively unimpaired. Results from this cohort are aligned with other studies suggesting that cognitively unimpaired individuals stand to lose the most after delirium.